# Matlab/R workflows to assess critical choices in Global Sensitivity Analysis using the SAFE toolbox

**DOI:** 10.1016/j.mex.2019.09.033

**Published:** 2019-09-30

**Authors:** Valentina Noacco, Fanny Sarrazin, Francesca Pianosi, Thorsten Wagener

**Affiliations:** a Department of Civil Engineering, University of Bristol, Bristol, BS8 1TR, UK; b Department of Computational Hydrosystems, UFZ-Helmholtz Centre for Environmental Research, 04318, Leipzig, Germany; c Cabot Institute, University of Bristol, Bristol, BS8 1UJ, UK

**Keywords:** Global Sensitivity Analysis, Earth system modelling, Sensitivity analysis, Input variability, Reproducibility, Uncertainty analysis, Output metric, Sample size, Simulation performance, Screening, Input interactions

## Abstract

Global Sensitivity Analysis (GSA) is a set of statistical techniques to investigate the effects of the uncertainty in the input factors of a mathematical model on the model’s outputs. The value of GSA for the construction, evaluation, and improvement of earth system models is reviewed in a companion paper by Wagener and Pianosi (2019). The present paper focuses on the implementation of GSA and provides a set of workflow scripts to assess the critical choices that GSA users need to make before and while executing GSA. The workflows proposed here can be adopted by GSA users and easily adjusted to a range of GSA methods. We demonstrate how to interpret the outcomes resulting from these different choices and how to revise the choices to improve GSA quality, using a simple rainfall-runoff model as an example. We implement the workflows in the SAFE toolbox, a widely used open source software for GSA available in MATLAB and R.

• The workflows aim to contribute to the dissemination of good practice in GSA applications.
• The workflows are well-documented and reusable, as a way to ensure robust and reproducible computational science.

## Graphical abstract

**Figure:**
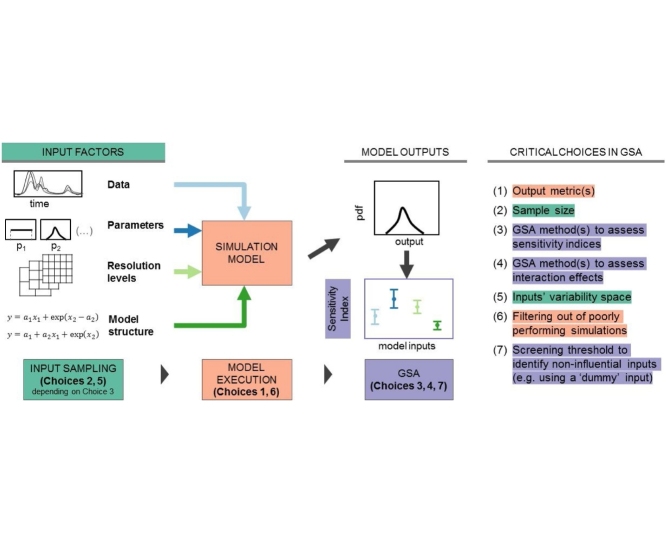

**Specification Table**

**Table:** 

Subject Area:	*Earth and Planetary Sciences*
More specific subject area:	*Uncertainty and Sensitivity Analysis in Earth System Modelling*
Method name:	*Global Sensitivity Analysis*
Name and reference of original method:	*Wagener and Pianosi et al. (2019)* [1]
Resource availability:	*SAFE toolbox available at:*www.safetoolbox.info

## Method details

Global Sensitivity Analysis (GSA) is a set of statistical techniques that allow to assess the effects of the uncertainty and variability in the input factors of a mathematical model on the model’s output(s) [2]. GSA has been shown to improve the construction and evaluation of earth system models and to maximise the information content that is extracted from model predictions [1]. The application of GSA involves running Monte Carlo simulations and some post-processing of input-output samples. The latter typically consists of the calculation of a set of sensitivity indices for the different input factors of the model. GSA users need to make a number of choices to set-up their GSA application. All these choices can significantly affect the GSA results, i.e. the estimated sensitivity indices, the consequent ordering of the most influential input factors (‘ranking’ in the GSA jargon), and the identification of non-influential input factors (‘screening’). Therefore, a thorough monitoring of the choices made is crucial to ensure the transparency and reproducibility of GSA results.

In the last decade the topic of reproducibility of scientific results has gained a growing interest, and concerns have been raised that a substantial number of scientific papers were falling short of this standard [3,4]. In particular, several papers (e.g. [[5], [6], [7]]) have stressed the importance of transparency and reproducibility in computational science to gain trust in the robustness of scientific results. Nonetheless, especially for large-scale, computationally expensive and time-consuming studies, the reproducibility quest might be difficult to achieve. One way to overcome this problem is to share the code that was developed to produce scientific results, along with well-documented and reusable workflows. The latter should combine the code and the data to produce the published scientific results [6].

In this paper, we will implement several GSA techniques by means of the SAFE (Sensitivity Analysis For Everybody) toolbox, which is available as Matlab code (SAFE version R1.1) and as an R package (SAFER version R1.0) [8]. A Python version of the SAFE toolbox is also currently under development. SAFE is an open source software which has been adopted so far by over 1700 academic users worldwide (by November 2018). The SAFE toolbox includes workflow scripts to guide users in applying GSA. The added value of the present paper is to provide workflows which make explicit the range of choices one should consider before and while running GSA, and to show the implications of different choices through practical examples. The rationale for the choices (‘remarks’) discussed in this paper is described in Section 2 of [1]. The workflows shown here aim to provide a basis for good practice and will help GSA users in their reproducibility quest. In fact, these workflows are generic and easy to customise. In turn, this will help in producing more transparent and robust GSA results, as the choices of the GSA users will be made explicit and the implications of these choices explored.

For illustration purposes, this paper uses the rainfall-runoff model HyMod, introduced by [9] and described in [10]. The model takes time series of precipitation and potential evapotranspiration as input and produces a time series of streamflow predictions as output. It includes five parameters: two parameters which control the soil moisture component and therefore the water balance (‘beta’ and ‘Sm’), and three parameters which control the routing component and therefore the streamflow dynamics (‘alfa’ which partitions the flow between slow and fast reservoirs, and ‘Rs’ and ‘Rf’ which define the residence time of the slow and fast flow reservoirs, respectively). The application study site is the Leaf River catchment, a 1950 km^2^ catchment located north of Collins, Mississippi, USA and described in [11].

Following this introduction, we provide an introductory code that sets-up the case study. Secondly, we discuss six critical choices in GSA applications. We provide below the code in Matlab/Octave and in the Supplementary material in R. The introductory code reports the basic steps that are necessary to generate the input-output samples that will be used in the subsequent remarks. Each remark can be run independently, but the order of the remarks follows what the authors believe is the natural sequence of choices GSA users would make in their analysis. For each remark, we report the code used to perform the analysis and the main figure showing the results, with a brief explanation of the results and their implications.

### Introductory code to run before any subsequent analysis

**Figure:**
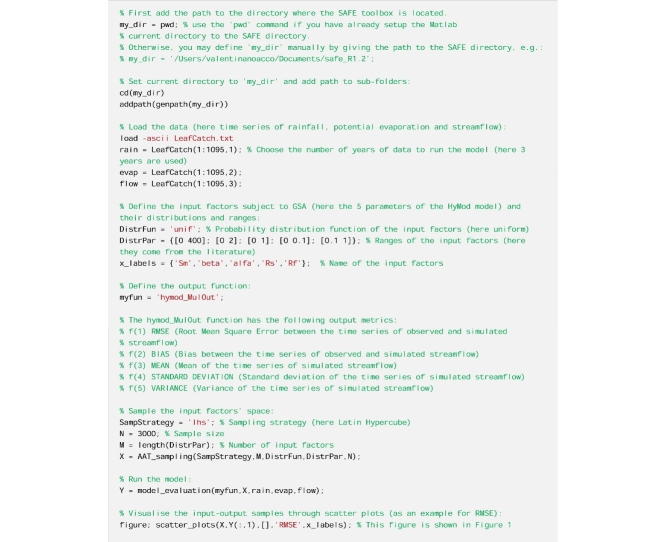

See Fig. 1

**Fig. 1:**
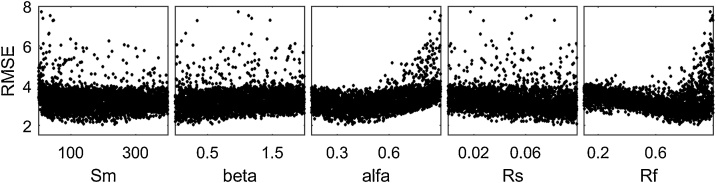
Scatter plots of the output samples (RMSE of streamflow predictions) against the five input factors (the 5 model parameters: maximum soil moisture (Sm [mm]), exponent in the soil moisture routine (beta [−]), partition coefficient between fast and slow flow routing (alfa [-]), coefficient of slow reservoir residence time (Rs [1/dd]) and coefficient of fast reservoir residence time (Rf [1/dd])). Roughly uniformly scattered points (e.g. Sm) indicate low sensitivity, while clear patterns when moving along the horizontal axis (e.g. alfa) denote higher sensitivity.

### Remark 1: Multiple definitions of the model output are possible

#### The problem

GSA assumes that the output metric is a scalar output. Nonetheless most earth system models yield time- and/or space- distributed outputs, which then need to be summarised in a scalar output metric for the purpose of the GSA, such as a performance metric or a statistic of the simulated variables. A choice of which scalar output metric to use is then required. Therefore, it is important to analyse the impact that different choices have on the GSA results. This remark is discussed in section 2.1 of [1].

#### Implementation details

To illustrate this point, we use the Regional Sensitivity Analysis (RSA) method, also called Monte Carlo filtering [2], and first proposed by [12] and [13]. We use the implementation of RSA based on grouping, introduced in [10], where the output values are ranked and divided into a prescribed number of groups (here ten), each with equal number of output samples. For each group, the corresponding Cumulative Distribution Function (CDF) is derived for each input. Then, for each input, a sensitivity index that measures the difference between the CDFs for the various groups is derived. In the analysis below we compute the maximum vertical distance between the CDFs (i.e. the Kolmogorov-Smirnov statistic [14,15]). In this example, four definitions of the model output are considered: two performance metrics (the root mean squared error, denoted as ‘RMSE’, and the absolute mean error, denoted as ‘bias’) and two statistics (the mean and the standard deviation of the simulated streamflow).

#### The code

**Figure:**
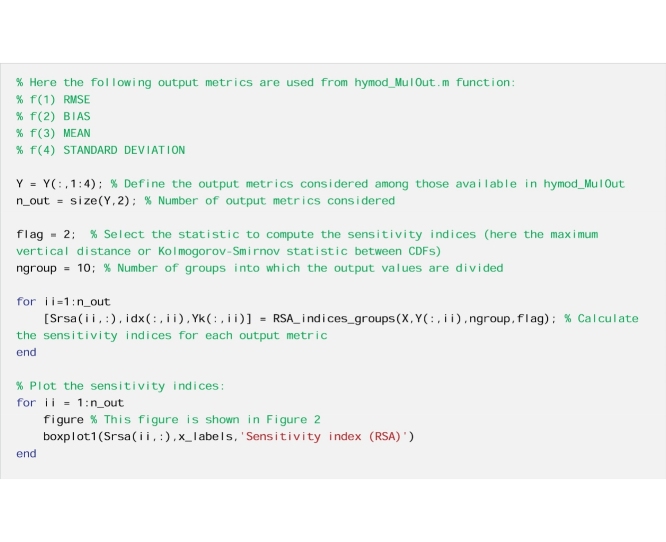

#### The results

**Fig. 2:**
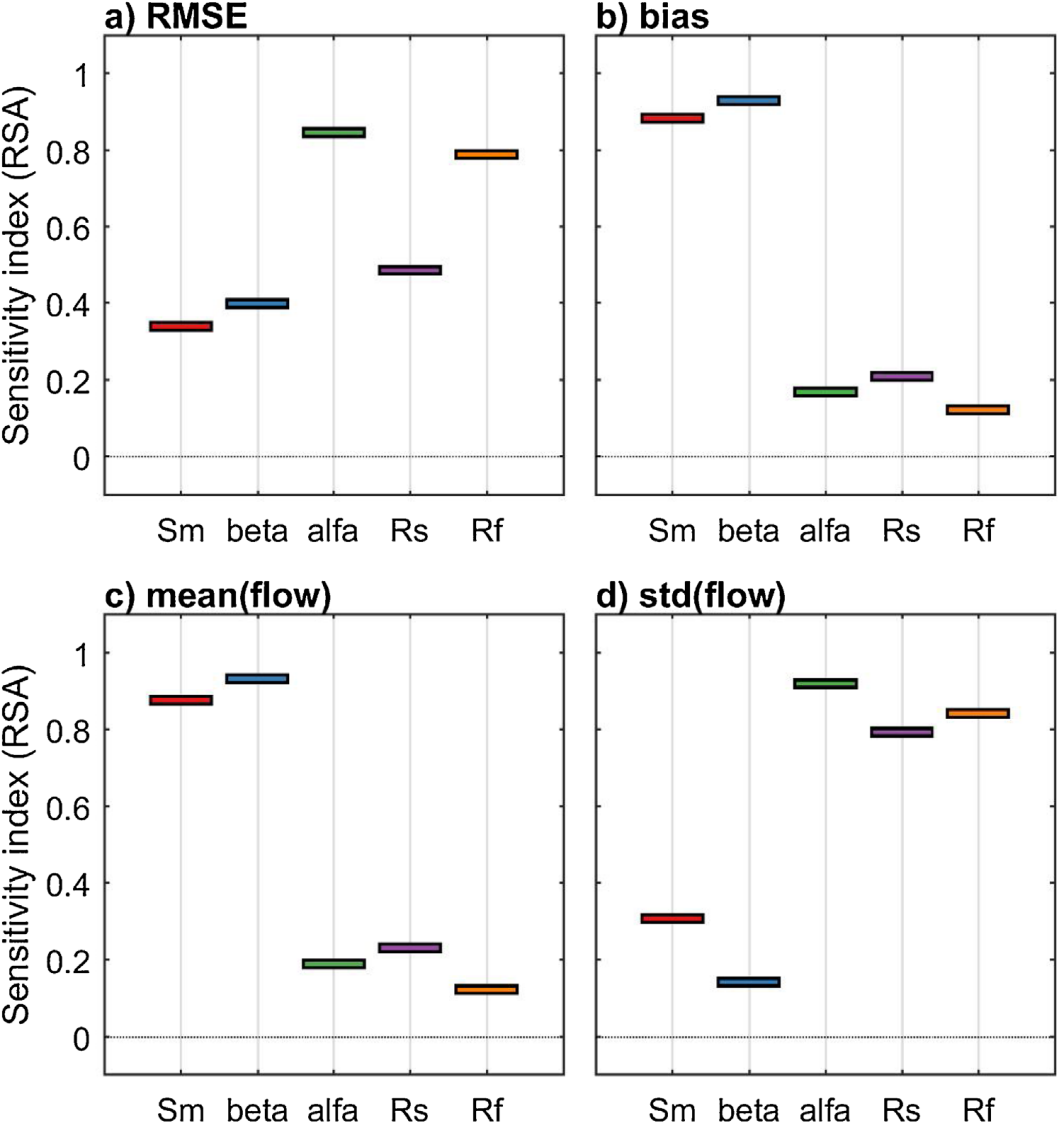
Sensitivity indices of four different output metrics: a) RMSE, b) bias, c) the mean of the simulated flow, d) the standard deviation (std) of the simulated flow.

From Fig. 2 we can see that different definitions of the output metric result in different ordering of importance of the input factors. For instance, RMSE and the standard deviation of the simulated flow are mainly influenced by alfa, Rs and Rf (Fig. 2a and d), while bias and the mean of the simulated flow are mainly influenced by Sm and beta (Fig. 2b and c). This is expected, because different model outputs capture different model behaviours: in this example, RMSE and the standard deviation of the simulated flow highlight the model dynamics (which are controlled by alfa, Rs and Rf), while bias and the mean of the simulated flow highlight the water balance (which are controlled by Sm and beta).

#### Interpretation of the results

**Fig. 3:**
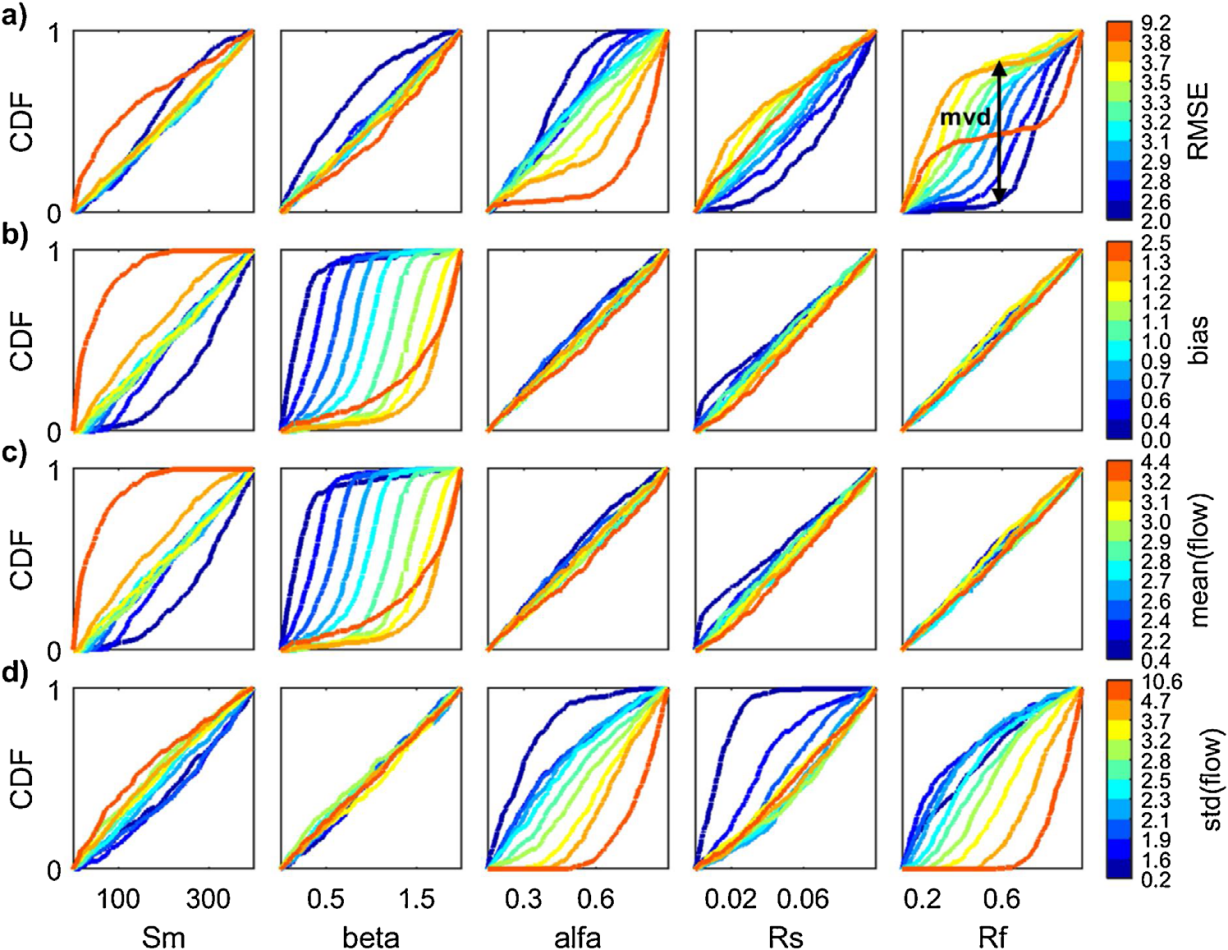
Cumulative Distribution Functions (CDFs) of the input factors for the four definitions of the output metric: a) RMSE, b) bias, c) mean(flow), d) std(flow). The sensitivity index for each input in Fig. 2 was derived as the maximum vertical distance (mvd) between the above CDFs of each input factor (see the arrow in the right plot of Fig. 3a). From this Figure we can see that the highest sensitivity of RMSE (a) and std(flow) (d) to parameters alfa, Rs and Rf is due to the larger spread between the CDFs of these input factors. A similar observation holds for bias (b) and mean(flow) (c), but in this case the largest spread among CDFs is that of input factors beta and alfa.

Now we look for an explanation of the results obtained by plotting the input factors' CDFs. The additional code used is reported below and Fig. 3 provides detailed explanations.

**Figure:**
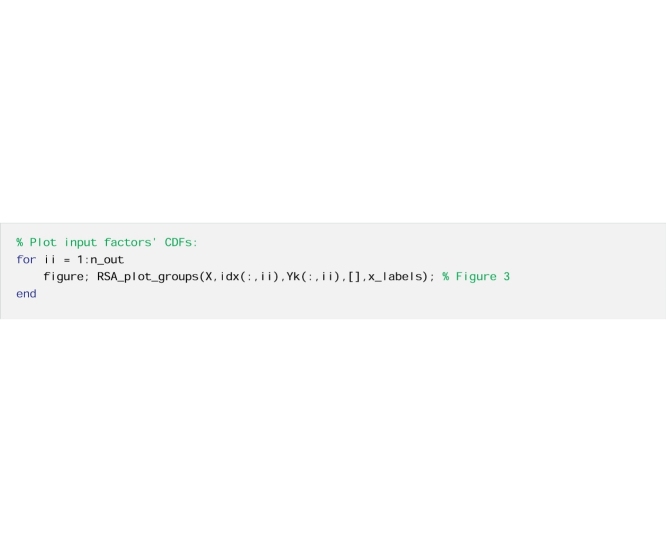

#### Implications

The choice of the definition of the model output should be based on the intended use of the model. Multiple definitions of the model output are advised, as they allow further insight to be gained into the model behaviour, as well as to check whether the model behaves as expected.

### Remark 2: Sample size also affects GSA results, so robustness of GSA results should be checked

#### The problem

The analytical computation of the sensitivity indices is generally not possible and sensitivity indices are typically approximated using an input-output sample obtained through Monte Carlo simulations. The choice of the sample size is therefore critical and should be evaluated so to find a good balance between the need for robust (i.e. sample independent) results and the need of limiting the computational cost of the analysis. In this section we demonstrate how to check that the sample size is large enough to obtain robust sensitivity analysis results, following the guidelines proposed in [16]. This remark is also discussed in section 2.5 of [1].

#### Implementation details

To illustrate this remark, we use the Elementary Effect Test (EET) [2], also called method of Morris [17]. The EET consists of calculating a number (r) of finite differences (also called Elementary Effects, EEs) for the different input factors at different points of the input factors’ space. The sensitivity index is then computed as the mean of the EEs across the input factors’ space. Contrary to RSA, the EET requires a tailored sampling strategy to calculate the sensitivity indices. Therefore, we cannot build on the samples drawn from the introductory code, and we need to derive a tailored input-output sample. In this application, the output metric is the performance metric RMSE.

#### The code

**Figure:**
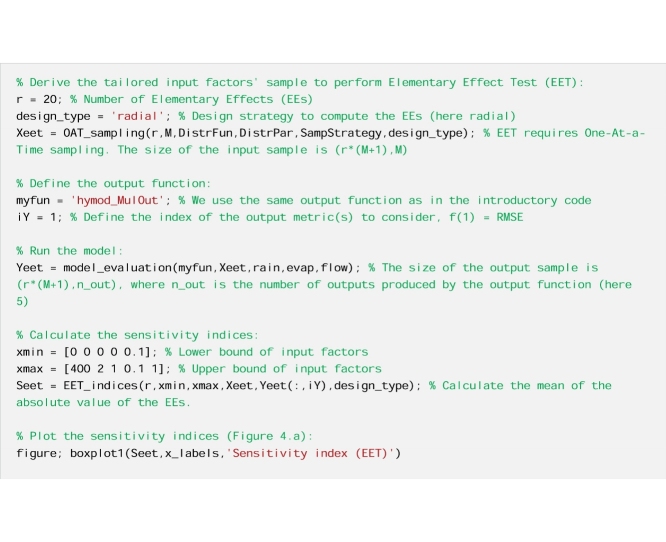

Now we repeat the estimation of the EET sensitivity indices using bootstrapping to derive confidence bounds around the sensitivity indices.

**Figure:**
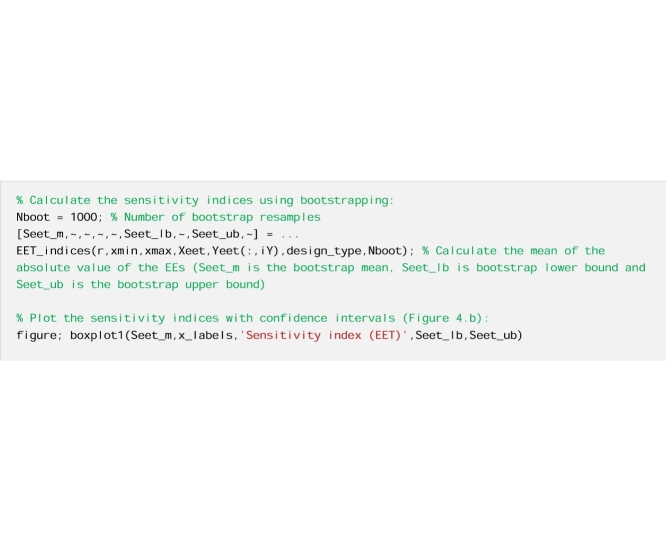

And finally, we repeat the calculations with a larger number of input-output samples.

**Figure:**
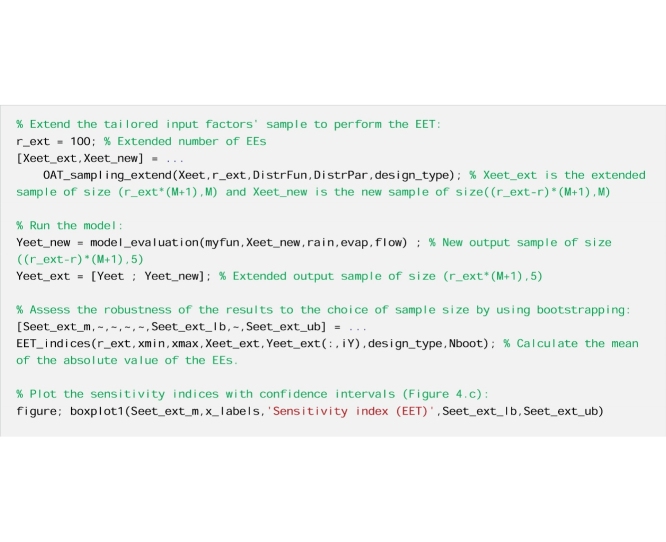

#### The results

**Fig. 4:**
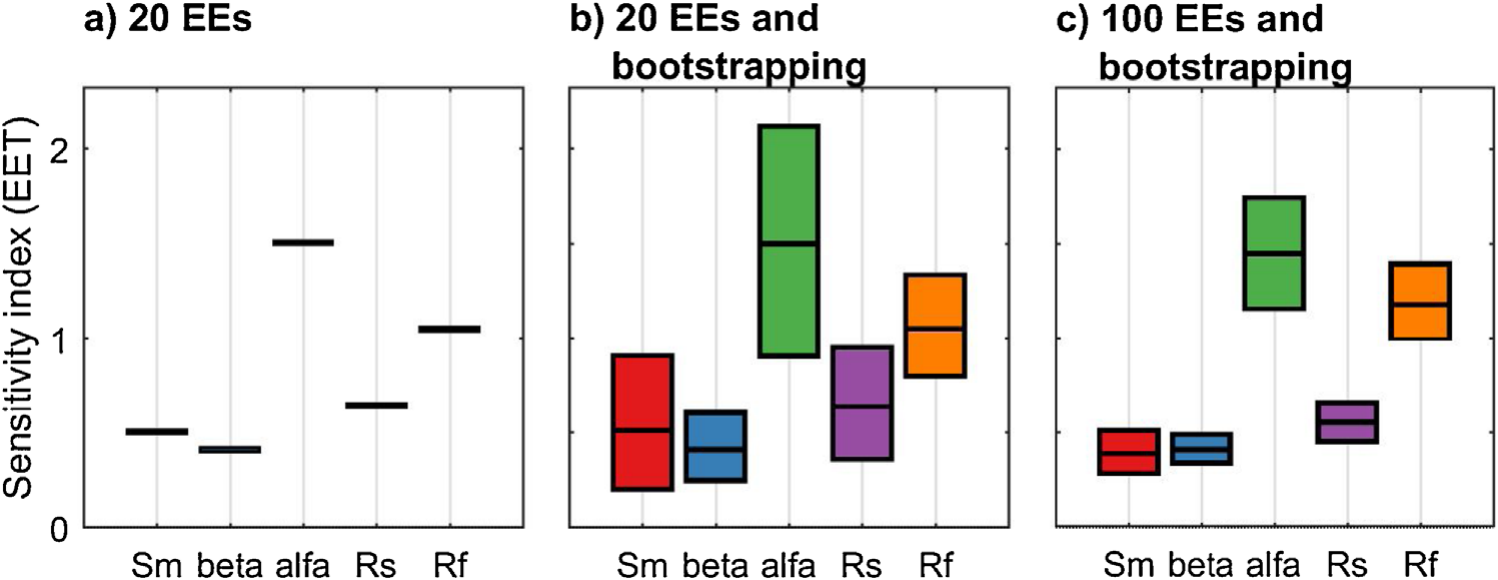
EET sensitivity indices (mean of EEs) computed using: a) r = 20 EEs, b) r = 20 EEs and bootstrapping, and c) r = 100 EEs and bootstrapping. In Fig. 4b–c the boxes represent the 95% bootstrap confidence intervals and the black lines indicate the bootstrap mean.

#### Interpretation of the results

By comparing Fig. 4a and b, which were both obtained using the same sample size (i.e. r = 20), we see the added value of deriving confidence bounds for the sensitivity indices. While Fig. 4a indicates that alfa and Rf are highly influential and Sm, beta and Rs are little influential, Fig. 4b shows that no robust conclusion can actually be drawn from the analysis because the confidence intervals of the input factors are overlapping. When we increase the sample size (Fig. 4c), we observe a reduction in the width of the confidence intervals, as expected, which means that the results become more robust to the choice of the sample size. We now see a clear separation between highly influential (i.e. alfa and Rf) and little influential (i.e. Sm, beta and Rs) input factors. We also note that in this specific example the ordering of the input factors does not change when increasing the sample size, however in general this may not be the case.

#### Implications

Assessing the robustness of the results via bootstrapping and visualising it through confidence intervals around the sensitivity indices is essential to check the robustness of the GSA results. The advantage of this method is that it does not require additional model executions. Wide and overlapping confidence intervals means that either the sample size should be increased if computationally affordable (as done in the above example), or the GSA user should opt for a less computationally demanding GSA method otherwise. The order of magnitude of the computational complexity of GSA methods is provided for instance in [18].

### Remark 3: Method choice matters as it can result in different sensitivity estimates (so using multiple methods is advisable)

#### The problem

Sensitivity indices can be computed using different GSA methods relying on different rationales and assumptions. This section shows that different GSA methods can result in different sensitivity estimates. This remark is discussed in section 2.3 of [1].

#### Implementation details

We apply two GSA methods, namely Variance-Based Sensitivity Analysis (VBSA) described in [19] and a moment-independent method, called PAWN and described in [20,21]. VBSA relies on the variance decomposition proposed by [22] and consists of assessing the contributions to the variance of the model output from variations in the input factors. In this example, we use as sensitivity index the first-order (main effect) index, which measures the variance contribution from variations in an individual input factor alone (i.e. excluding interactions with other factors). Similar to the EET, VBSA requires a tailored sampling strategy and therefore we need to derive a tailored input-output sample.

In contrast to the VBSA method, the PAWN method estimates the effect of the input factors on the entire output distribution, instead of on its variance only. The method compares the output unconditional CDF, which is obtained by varying all input factors simultaneously, to the conditional CDFs that are obtained by varying all input factors but one, which is restrained to a prescribed conditioning interval [21]. For each input factor, we use n (here ten) conditional CDFs and calculate the average Kolmogorov-Smirnov (KS) statistic [14,15] (i.e. average maximum vertical distance) between the *n* conditional CDFs and the unconditional one, using the code available at https://www.safetoolbox.info/pawn-method/. In this example, the output scalar metric is the variance of the simulated flow.

#### The code

We perform Variance-Based Sensitivity Analysis (VBSA).

**Figure:**
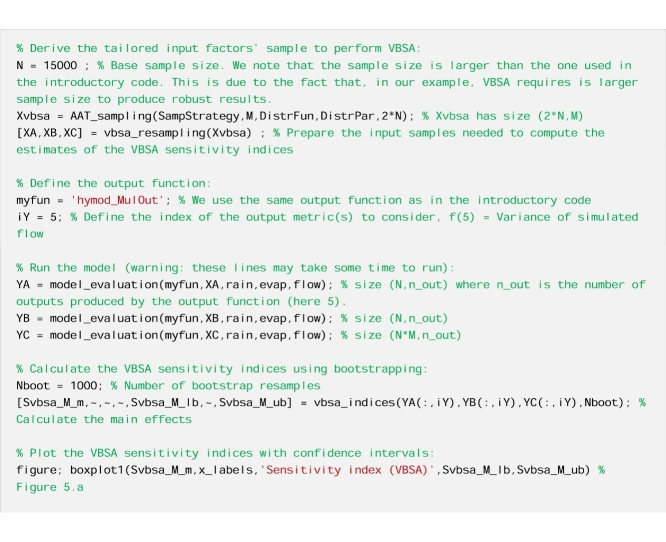

We perform PAWN.

**Figure:**
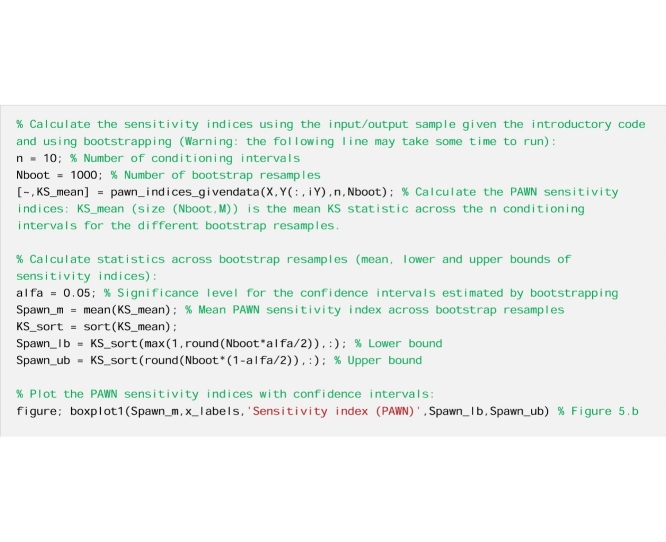

#### The results

**Fig. 5:**
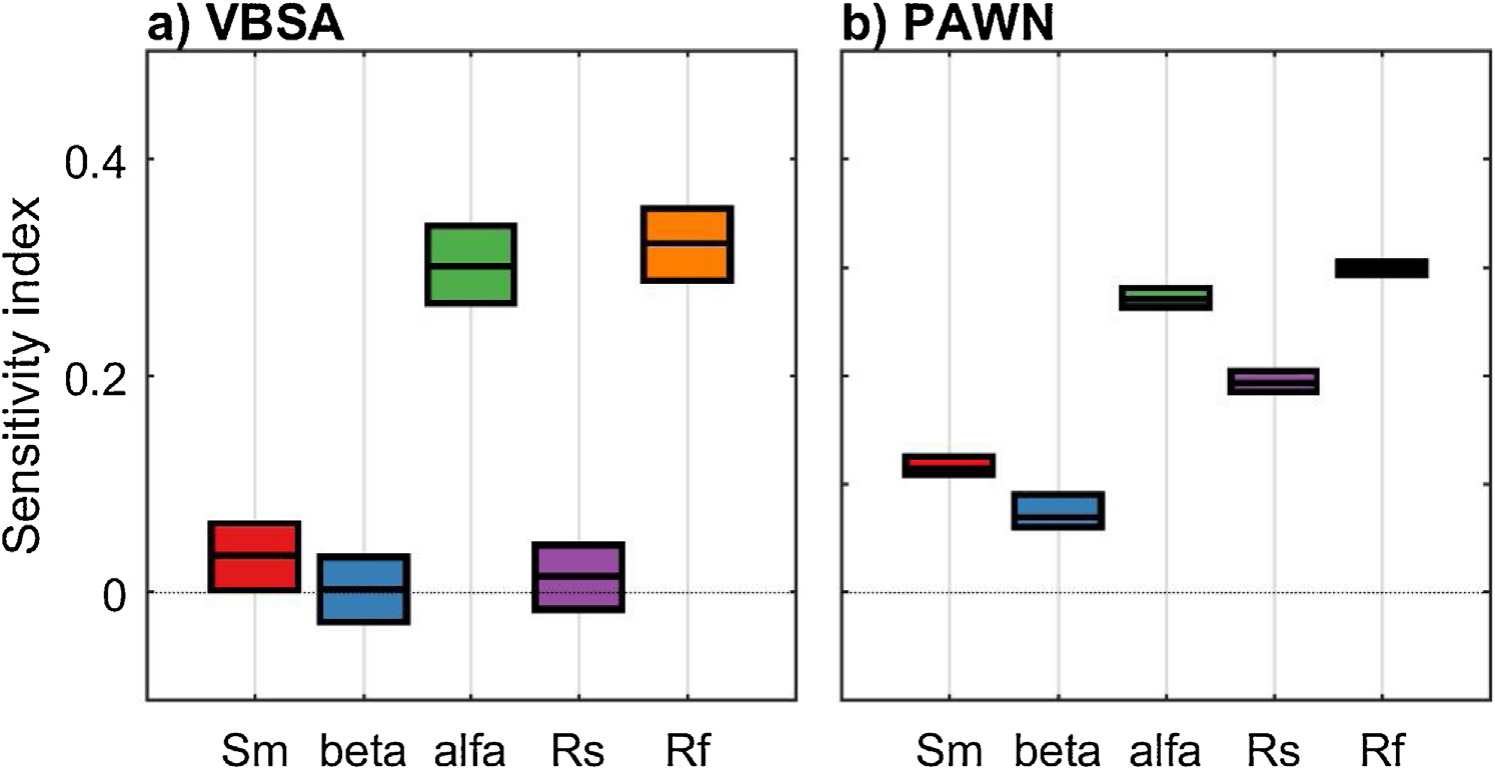
Sensitivity indices calculated using a) the VBSA methods (main effects) and b) the PAWN method (mean value of the KS statistic across the conditioning intervals). The boxes represent the 95% bootstrap confidence intervals and the black lines indicate the bootstrap mean.

From Fig. 5a, we see that Sm, beta and Rs have a similarly low value of the variance-based sensitivity index, with largely overlapping confidence intervals, while alfa and Rf have a much higher sensitivity index. By using PAWN (Fig. 5b), we still infer that alfa and Rf are the two most influential input factors, however Rs appears to be the third most influential input factor, with a distinctively higher sensitivity index than Sm and beta. The relative importance of Rs is thus judged quite differently depending on the GSA method used. Therefore, in the interpretation of the results we will focus on the parameter Rs, as an example.

#### Interpretation of the results

**Fig. 6:**
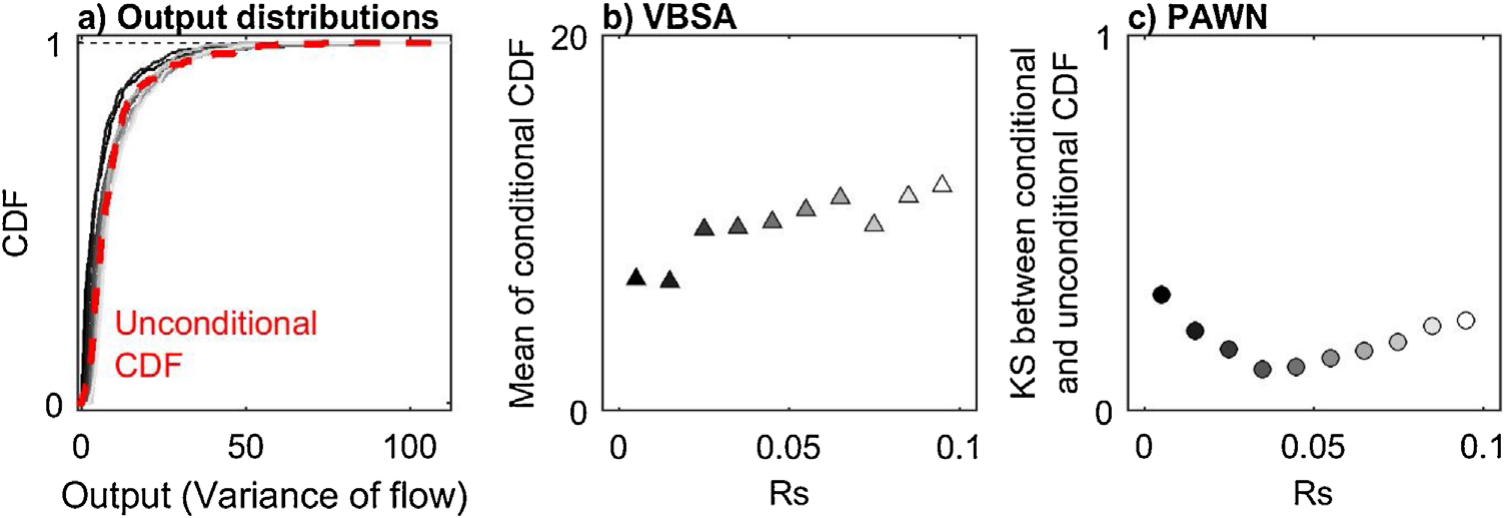
Distribution and statistics of conditional and unconditional outputs (variance of simulated flow) for parameter Rs. a) Cumulative Distribution Functions (CDFs) of unconditional output (red dashed line) and of conditional outputs obtained by fixing the value of Rs within one of the ten conditioning intervals (grey lines). b) Mean of conditional outputs for the ten conditioning intervals (grey triangles). c) KS statistic between unconditional and conditional CDFs for the ten conditioning intervals (grey circles).

Now we look for an explanation of the results obtained by examining the conditional and unconditional output distributions. The additional code used is reported below and Fig. 6 provides detailed explanations.

**Figure:**
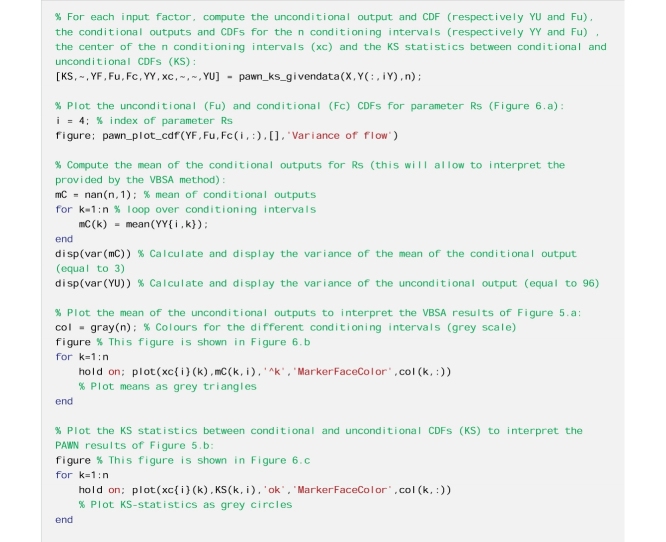

From Fig. 6a, we observe differences between the unconditional CDF and the conditional CDFs, and in particular over the lower range of values of the output (i.e. variance of flow below 10). As a result, the KS statistics shown in Fig. 6c take high values, which explains the large value of the PAWN sensitivity index (Fig. 5b). On the contrary, from Fig. 6b, we observe little variation in the mean of the conditional CDFs across the conditioning intervals (values are between 6.9 and 12). We further estimate that the variance of the mean of conditional CDFs across the conditioning intervals is equal to 3, which is very small compared to the variance of the unconditional CDF which is equal to 93. This means that the contribution of Rs to the total output variance is very small. This explains the low value of the VBSA sensitivity index (Fig. 5a).

#### Implications

The choice of the GSA method is critical as different methods focus on different aspects of the model input-output response and may therefore lead to different sensitivity estimates. In the example examined in this remark, the two methods used (VBSA and PAWN) analyse different aspects of the output distribution. This leads to slightly different conclusions, where the same parameter is considered uninfluential when only looking at the output variance (VBSA), and relatively influential when considering the entire output CDF (PAWN). It is also known that the different GSA methods have different ability to capture interactions among input factors as explained for instance in [2]. Interactions are further analysed in remark 4. Consequently, we advise that the choice of GSA method should depend on the objective of the analysis and, when possible, that the GSA user should aplply different methods to the same case study for a more exhaustive analysis of the model sensitivity. The task is facilitated by the increasing availability of sensitivity approximation techniques that can be applied to the same generic input-output sample (e.g. [23]).

### Remark 4: GSA methods can measure direct and joint effects of input factors across their variability space

#### The problem

In complex dynamic models such as earth system models, input factors often have a high level of interactions, which means that the effect of a given input factor on the output(s) can be amplified or reduced depending on the value taken by the other input factors. Some GSA methods, such as Variance-Based Sensitivity Analysis introduced in remark 3, allow to capture input factors’ interactions. This remark is discussed in section 2.2 of [1].

#### Implementation details

We calculate three sensitivity indices using the VSBA methods, i.e. (1) the main effect index (used in remark 3), which measures the direct effect of each input factor, (2) the total effect index, which measures the direct effect and the interaction effects with all other input factors and (3) the interaction effect index, which is the difference between total and main effect index. We use the same output metric and input-output samples generated in remark 3.

#### The code

We compute the main and total effect sensitivity index using VBSA.

**Figure:**
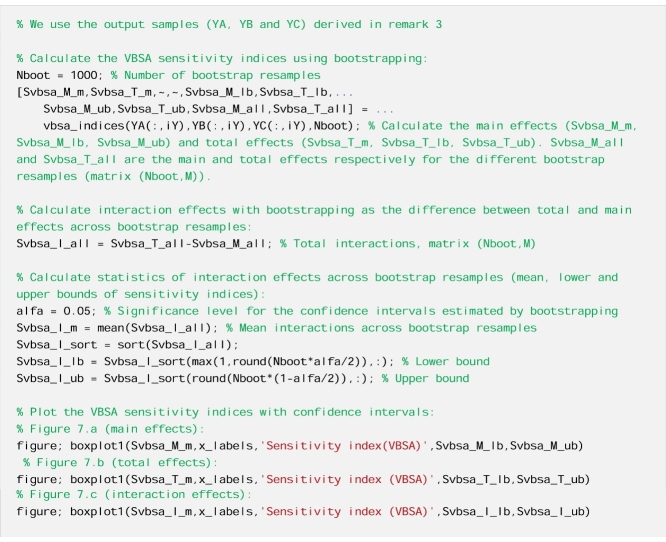

#### The results

**Fig. 7:**
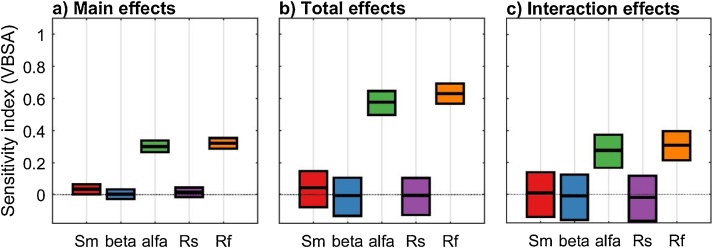
Sensitivity indices calculated using the VBSA methods a) main effects indices, b) total effect indices and c) total interaction effect indices. The boxes represent the 95% bootstrap confidence intervals and the central black lines indicate the bootstrap mean.

We observe that the total effect sensitivity index of parameters alfa and Rf (Fig. 7b) is significantly higher than the main effect sensitivity index (Fig. 7a). This means that these two parameters have an effect on the output not only through their individual variations but also through interactions, as we can see from Fig. 7c which shows the total interaction effect index. Instead, the other three parameters (Sm, beta and Rs) have low main, total and interaction effect indices, and therefore these three parameters have a small effect, both direct and through interactions.

#### Interpretation of the results

**Fig. 8:**
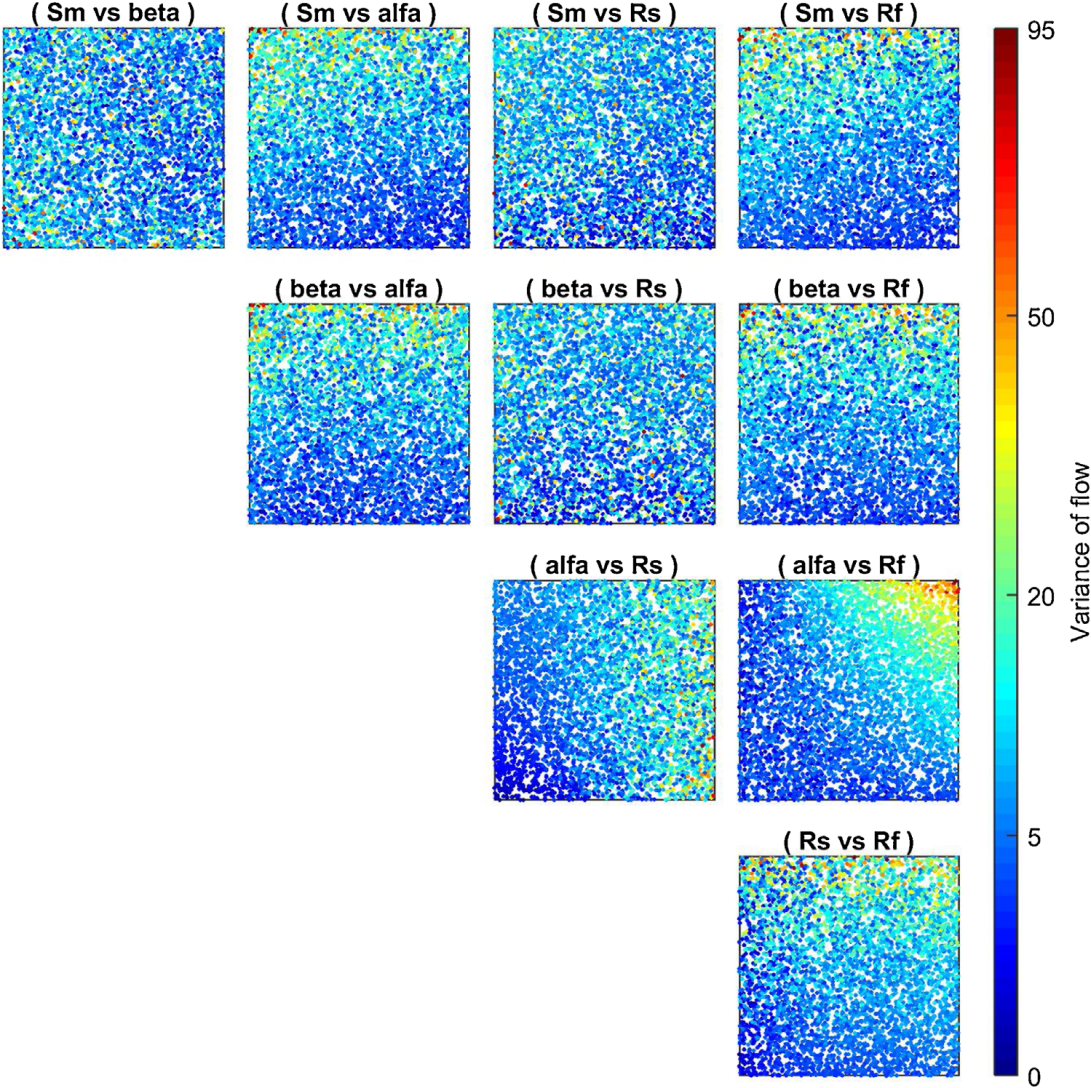
Two-dimensional scatter plots. The colour indicates the value of the output (Variance of flow). The plot (alfa vs Rf) shows that high values of the variance (red colour) are obtained only when both alfa and Rf have values close to the upper bound of their range of variability. This means that these two input factors are interacting. On the contrary the other scatter plots do not show marked patterns and therefore the second order effects for the other pairs of input factors appear to be small.

Now we look for an explanation of the results by examining the two-dimensional scatter plots that allow to identify pairwise interactions (second order effects) between input factors. The additional code used is reported below and Fig. 8 provides detailed explanations.

**Figure:**
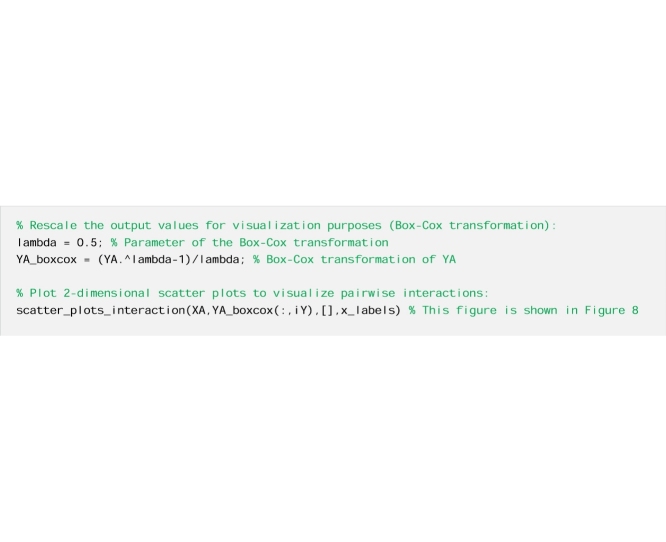

#### Implications

It is important to use a GSA method that can detect parameter interactions, such as VBSA, if this information is relevant. In addition, as discussed in [24], scatter plot are easy-to-implement tools that can complement the results of a rigorous and quantitative GSA method and that provide insight into pairwise interactions between input factors. Nonetheless, complex interactions might not be visible from a scatterplot, that is why using VBSA, coupled with other methods, might be useful to highlight interactions between input factors.

### Remark 5: The definition of the space of variability of the input factors has potentially a great impact on GSA

#### The problem

Sampling the input factors’ space requires the definition of the distribution and range of the input factors. This section shows how the choice of the range of the input factors can impact the sensitivity indices. This remark is discussed in section 2.4 of [1].

#### Implementation details

We use RSA as in remark 1, but here we adopt the variant of RSA with threshold, where the input samples are divided into two groups (‘behavioural’ and ‘non-behavioural’), whether their corresponding output falls above or below a prescribed threshold [12,13]. The output metric selected is the RMSE.

#### The code

**Figure:**
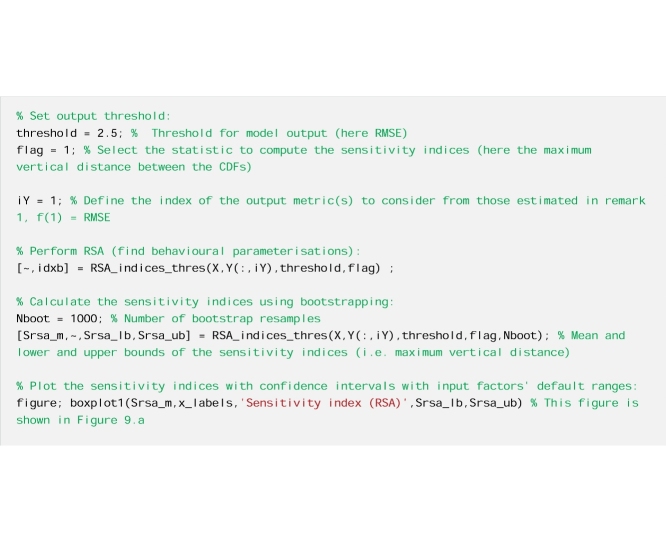

Now we repeat the estimation of the RSA sensitivity indices using modified input factors' ranges.

**Figure:**
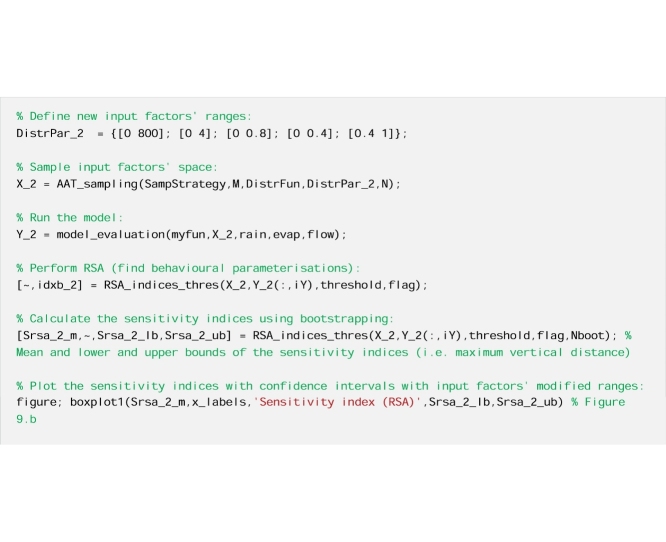

#### The results

**Fig. 9:**
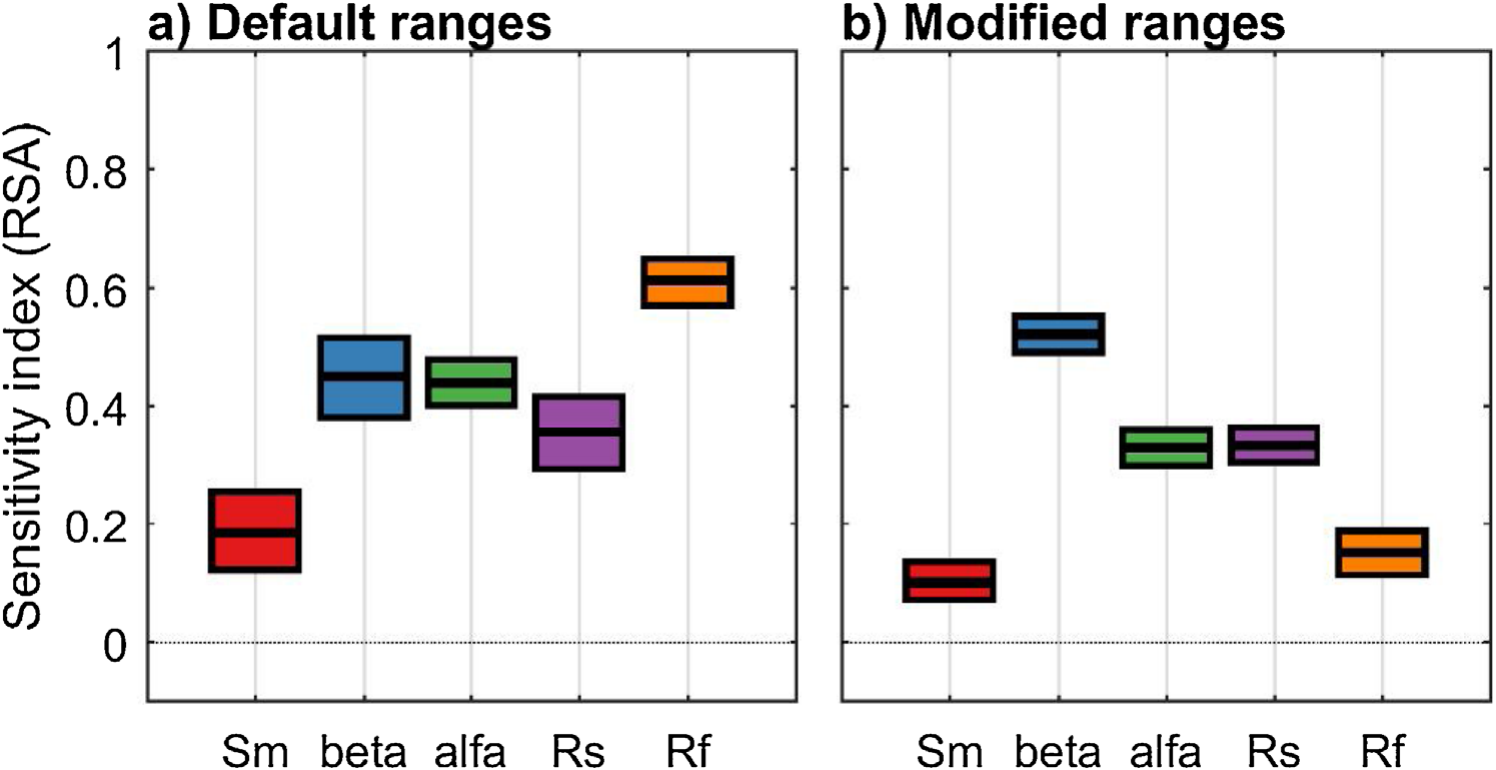
Sensitivity indices calculated using a) the default ranges of the input factors taken from the literature and b) modified ranges. The boxes represent the 95% bootstrap confidence intervals and the central black lines indicate the bootstrap mean.

The results reported in Fig. 9 show that changing the ranges of the input factors can change the sensitivity indices, as expected. In the specific, decreasing the range of Rf decreases its sensitivity index considerably, while decreasing the range of alfa slightly decreases its sensitivity index, and increasing considerably the range of Sm only slightly decreases its sensitivity index.

#### Interpretation of the results

**Fig. 10:**
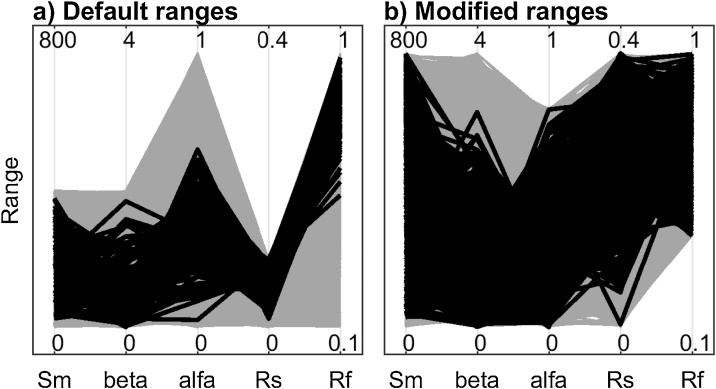
Parallel coordinate plots representing the behavioural parameterisations (black lines) and the non-behavioural ones (grey lines) for the analysis with a) default input factors’ ranges and b) modified ranges. In the case with modified ranges, we decreased the range of Rf. We see that the lower part of the default range of Rf provides non-behavioural parameterisations only (Fig. 10a), while both behavioural and non-behavioural parameterisations can be found throughout the reduced range of Rf (Fig. 10b). This explains the higher sensitivity index of Rf with the default ranges (Fig. 9a) compared to the modified ranges (Fig. 9b). Similarly, reducing the range for alfa (in this case decreasing its upper bound) has the effect of decreasing the sensitivity index of alfa (as shown in Fig. 9). In fact, alfa has most of its behavioural samples in the lower range and therefore the difference between behavioural and non-behavioural is reduced (as shown in Fig. 10). Fig. 10 allows analysis of the parameter ranges over the behavioural and non-behavioural parameter, thus explaining the direction of change in the sensitivity index of alfa and Rf shown in Fig. 9. For an explanation of the magnitude of change in the sensitivity indices, one could further analyse the Cumulative Distribution Functions of the parameters over the behavioural and non-behavioural parameter sets.

Now we look for an explanation for the direction of change for the modified ranges by examining the ranges of the behavioural parameterisations using parallel coordinate plot. The additional code used is reported below and Fig. 10 provides detailed explanations.

**Figure:**
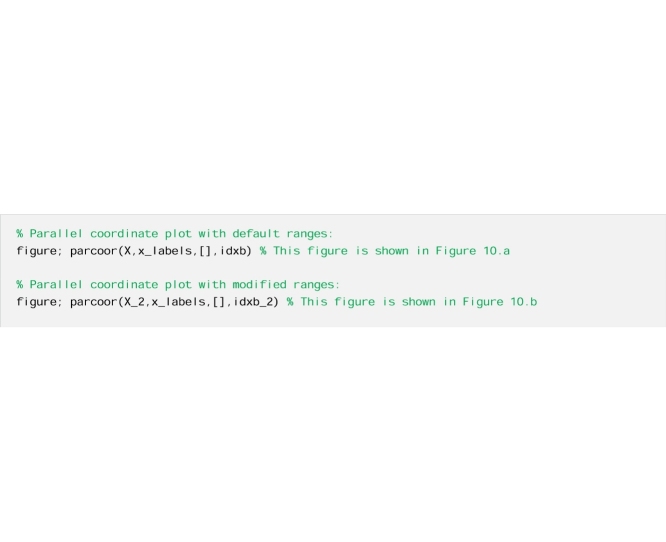

#### Implications

Changing the range of variability of an input factor can greatly change its sensitivity index. Therefore, careful consideration should be given to choose a range wide enough so that it includes all feasible (physical) values according to expert knowledge or literature values, but not too wide so that it excludes infeasible values. In this remark we varied the ranges of the input factors only (we used a uniform distribution). The effect of different distributions on the input factors’ sensitivity estimates could also be tested, especially if a priory information is available on their distributions. Another implication is that sensitivity analysis and performance analysis go hand in hand, as discussed in greater detail in the next remark.

### Remark 6: It may be necessary to remove poorly performing simulations to avoid that they dominate the estimation of sensitivity indices

#### The problem

Adopting input factors ranges that are too wide might result in the inclusion of poorly performing input factors values, which in turn might affect the sensitivity analysis results, as shown in remark 5. In this section, we analyse the effect of filtering out poorly performing input factors’ sets based on their corresponding output values. We show the importance of running sensitivity analysis with and without the inclusion of poorly performing (sometimes called non-behavioural) simulations to assess their impact on the results. This remark forms part of the discussion in section 2.4 of [1].

#### Implementation details

We use RSA as in remark 1, i.e. with the implementation of RSA based on grouping. The output values are again divided into ten groups and the non-behavioural simulations are set to be those related to the highest (or lowest) performance metric (here set to be those with RMSE > 3.8, i.e. the group with the least performing simulations according to the performance metric chosen).

#### The code

We run RSA based on grouping as in remark 1, but here the confidence intervals of the sensitivity indices are also estimated with bootstrapping.

**Figure:**
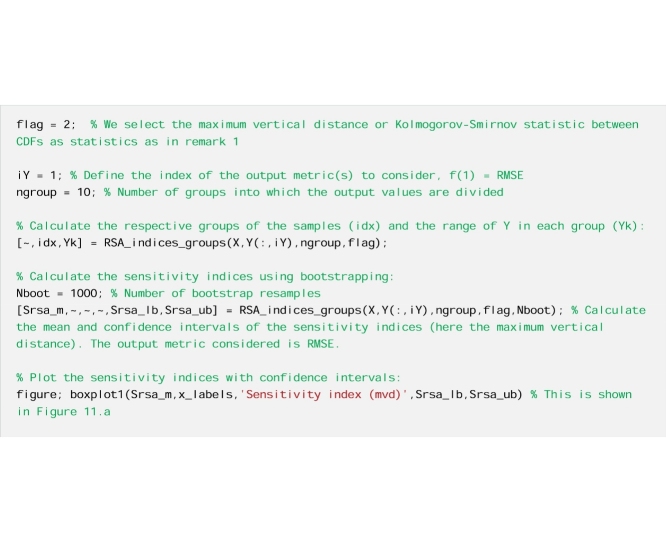

Now we remove the poorly performing simulations and rerun the analysis. First, we decide the value of the performance metric above (or below) which simulations are considered poorly performing

**Figure:**
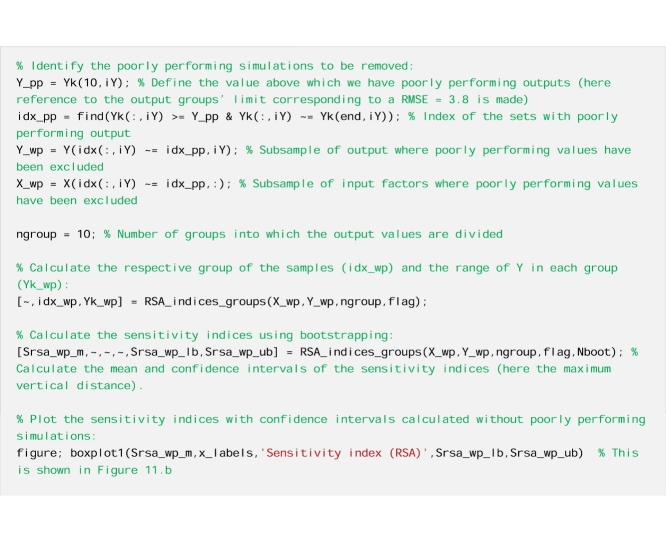

#### The results

**Fig. 11:**
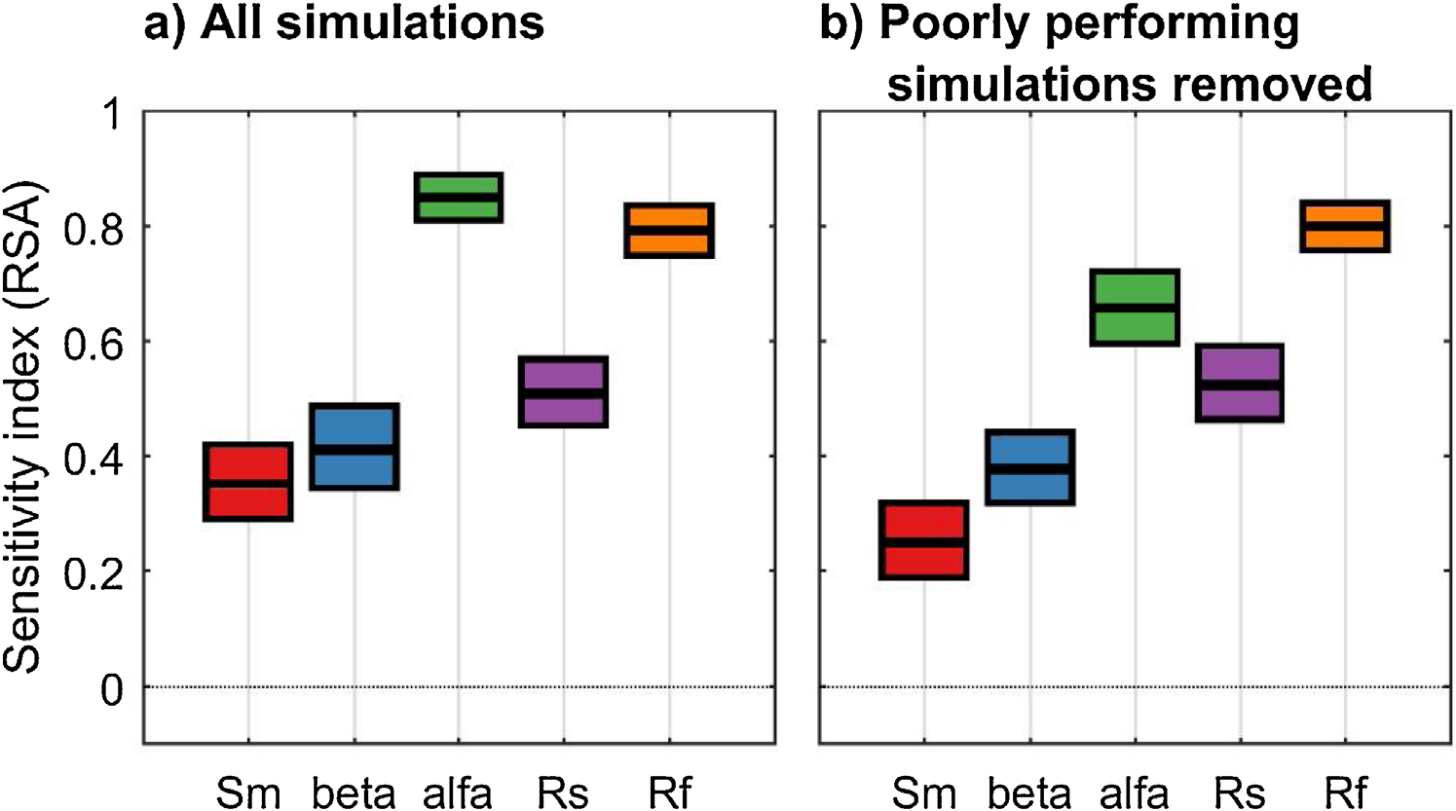
Sensitivity indices calculated a) with all the simulations and b) with poorly performing simulations removed. The boxes represent the 95% bootstrap confidence intervals and the central black lines indicate the bootstrap mean.

The results reported in Fig. 11 show that removing the poorly performing simulations can change the sensitivity indices, as expected. In this specific case, it leads to a decrease in the sensitivity index of alfa.

#### Interpretation of the results

**Fig. 12:**
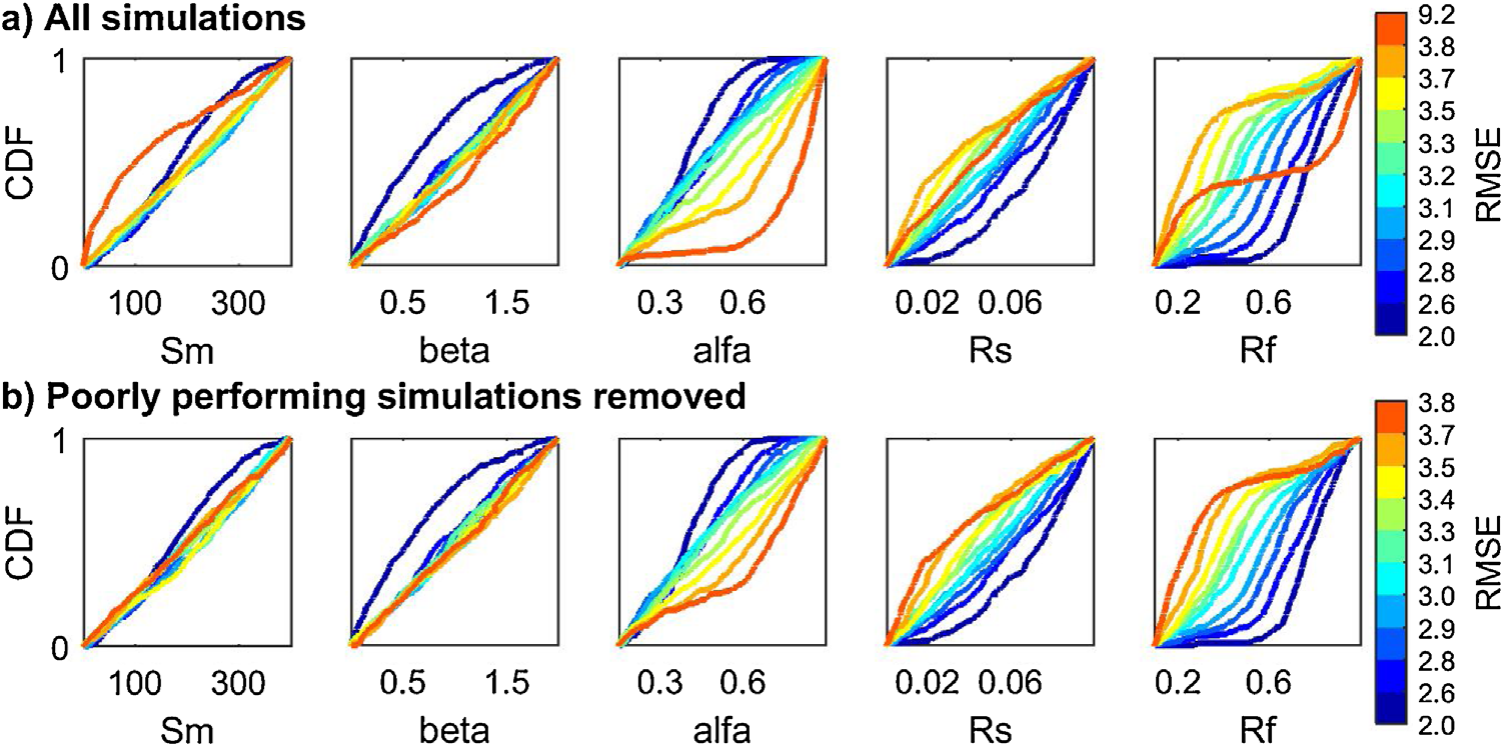
Cumulative Distribution Functions (CDFs) of the input factors: a) when using all the simulations, b) when removing the poorly performing simulations. The sensitivity index for each input in Fig. 11 was derived as the maximum vertical distance (mvd) between the above CDFs (as explained in Fig. 3). We can see that the poorly performing simulations in Fig. 12a (i.e. with RMSE > 3.8, red CDF) have been removed in Fig. 12b. This has the effect of decreasing the mvd for alfa as it eliminates the red CDF of Fig. 12a which stands out from the other CDFs for these two input factors. In fact, for alfa, the red CDF of Fig. 10a shows that most of the worst performing samples of alfa are in its upper range. Instead, for example for Rf, the removal of poorly performing simulations (red CDF in Fig. 12a) does not affect the mvd.

Now we look for an explanation of the results obtained by plotting the input factors' CDFs. The additional code used is reported below and Fig. 12 provides detailed explanations.

**Figure:**
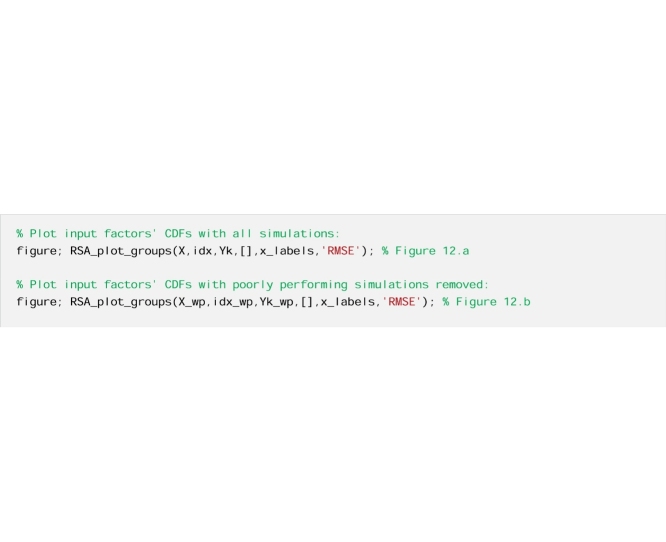

#### Implications

Including poorly performing simulations can impact the results of the sensitivity analysis and might change how influential the input factors actually are. Therefore, an analysis of the subranges of values of the input factors which give poorly performing simulations should be carried out. The modeller should explicitly consider whether these subranges should be included in the sensitivity analysis or not.

### Remark 7: Influential and non-influential input factors can be identified using a ‘dummy’ input factor

#### The problem

GSA is commonly applied to identify the non-influential input factors that can be set to any value within their space of variability with negligible effect on the output [2]. To set a threshold value on the sensitivity indices to separate influential and non-influential input factors (i.e. a screening threshold) [25], proposed to artificially introduce an additional ‘dummy’ input factor. Such a ‘dummy’ input factor is defined to have no impact on the output because it does not appear in the model equations. In this remark, we demonstrate the use of the ‘dummy’ input factor to screen influential and non-influential input factors. This remark forms part of the discussion in section 2.5 of [1].

#### The implementation

We use the PAWN method described in remark 3, because the method can be used for screening purposes [20]. As in remark 3, we apply PAWN to a generic input/output sample using the code available at https://www.safetoolbox.info/pawn-method/. This code also implements the computation of the PAWN sensitivity indices for the dummy input factor. We adopt as sensitivity index the maximum value of the KS statistic across the conditional CDFs. This sensitivity index is an appropriate metric to identify non-influential input factors (and therefore for screening purposes) as it allows to detect whether an input factor has an effect on the output for at least one conditioning interval. The sensitivity index for the dummy input factor estimates the error in approximating the ‘true’ CDFs using the current input/output sample. The output metric selected to perform PAWN is the bias.

#### The code

We perform PAWN using a subsample of the input/output sample derived in the introductory code, to determine whether we could use a smaller sample to screen the input factors.

**Figure:**
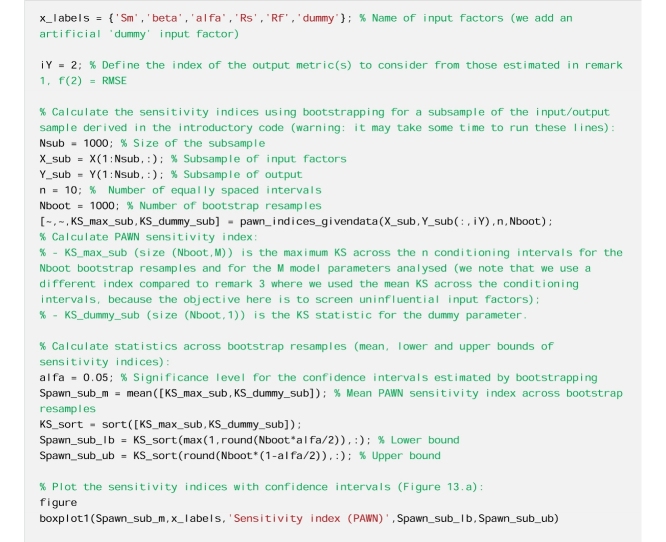

Now we repeat the calculation of the PAWN sensitivity indices using the entire input/output sample derived in the introductory code.

**Figure:**
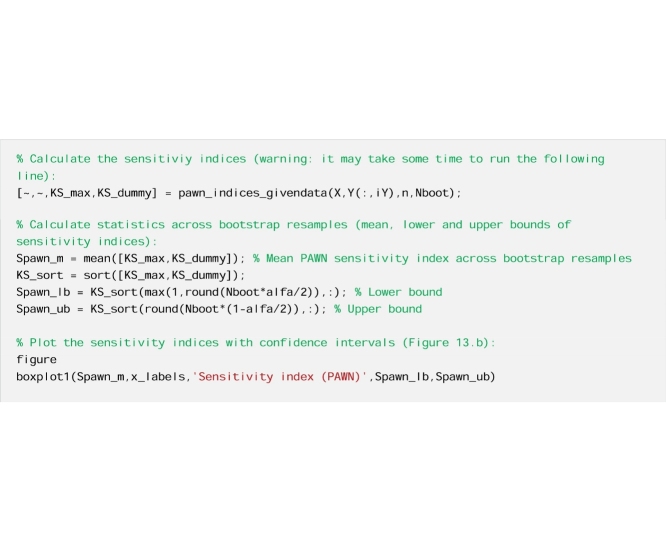

#### The results

**Fig. 13:**
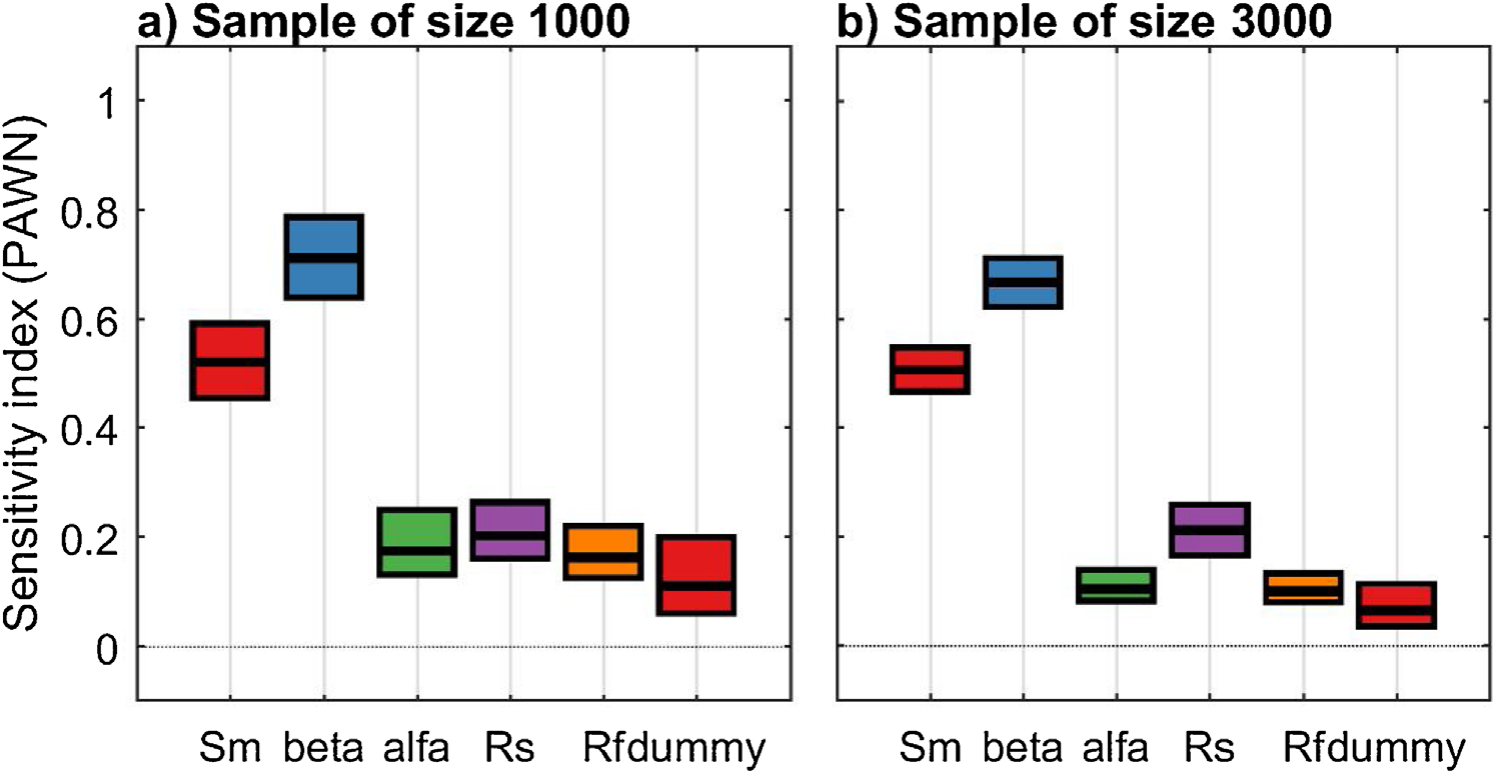
PAWN sensitivity indices for the five model input factors and the additional ‘dummy’ input factor, calculated using an input/output sample a) of size 1000 and b) of size 3000. The boxes represent the 95% bootstrap confidence intervals and the central black lines indicate the bootstrap mean.

#### Interpretation of the results

From Fig. 13a, we observe that the confidence intervals of the sensitivity index for three model input factors (alfa, Rs and Rf) are overlapping with the confidence intervals of the dummy input factor. On the other hand, the sensitivity index of Sm and beta are significantly higher than the sensitivity index of the dummy input factor. This means that, when using a sample of size 1000, we can conclude that Sm and beta are influential parameters, while alfa, Rs and Rf appear to be non-influential, because their sensitivity index is of the same order of magnitude as the approximation error of the CDFs. However, we observe that the confidence intervals on the sensitivity indices are rather wide, including for the low-sensitivity input factors. When using a larger sample size (Fig. 13b), we observe a significant reduction in the width of the confidence intervals, which results in a clear separation between Rs and the dummy input factor. This means that we can robustly infer that Rs is an influential input factor using a sample of size 3000 (Fig. 13b), while the sample of size 1000 was too small to robustly identify the non-influential input factors (Fig. 13a).

#### Implications

The method discussed in this remark identifies the influential input factors as those factors that have a sensitivity index (and corresponding confidence intervals) higher than, (and not overlapping with), the sensitivity index of the dummy input factor. Input factors that have a sensitivity index lower than (or overlapping with) the dummy input factor can be considered non-influential, because their sensitivity index has the same magnitude as the approximation error of the sensitivity indices. However, the choice of the sample size is critical to obtain robust screening results (see further discussion on the choice of the sample size in remark 3). Specifically, it can be expected that the number of non-influential input factors identified using the ‘dummy’ approach will decrease with increasing sample size, as the approximation error of the sensitivity indices reduces.

## Author contributions

V.N., F.S., F.P. and T.W. designed the study; V.N. and F.S. have contributed in equal measure to the preparation of the workflows and to the writing of the manuscript, with contributions from F.P. and T.W.; all authors have approved the final version of the manuscript.
